# Dynamic conjugate F-SHARP microscopy

**DOI:** 10.1038/s41377-020-00348-x

**Published:** 2020-06-30

**Authors:** Ioannis N. Papadopoulos, Jean-Sebastien Jouhanneau, Naoya Takahashi, David Kaplan, Matthew Larkum, James Poulet, Benjamin Judkewitz

**Affiliations:** 1 Charité – Universitätsmedizin Berlin, Einstein Center for Neurosciences, NeuroCure Cluster of Excellence, Charitéplatz 1, 10117 Berlin, Germany; 2 Max Delbrück Center for Molecular Medicine, Robert-Rössle-Str. 10, 13092 Berlin, Germany; 3 Institute for Biology, Humboldt University, Charitéplatz 1, 10117 Berlin, Germany

**Keywords:** Multiphoton microscopy, Interference microscopy

## Abstract

Optical microscopy is an indispensable tool in biomedical sciences, but its reach in deep tissues is limited due to aberrations and scattering. This problem can be overcome by wavefront-shaping techniques, albeit at limited fields of view (FOVs). Inspired by astronomical imaging, conjugate wavefront shaping can lead to an increased field of view in microscopy, but this correction is limited to a set depth and cannot be dynamically adapted. Here, we present a conjugate wavefront-shaping scheme based on focus scanning holographic aberration probing (F-SHARP). We combine it with a compact implementation that can be readily adapted to a variety of commercial and home-built two-photon microscopes. We demonstrate the power of the method by imaging with high resolution over extended FOV (>80 µm) deeper than 400 μm inside a mouse brain through a thinned skull.

## Microscopy: sharp, deep, wide images with F-SHARP

A new microscopic approach helps obtain sharp images, with a wide field of view, in deep tissues. Focus scanning holographic aberration probing (F-SHARP) was developed by Benjamin Judkewitz of Charité–Universitätsmedizin Berlin and colleagues in Germany. This microscopic scheme can be readily adapted to a variety of two-photon microscopes. The team used their method for imaging neurons in live mice brains through a thinned skull, providing images with a field of view of wider than 80 µm and to a depth of more than 400 µm. F-SHARP uses wavefront shaping and a spatial light modulator to correct the aberrations and scattering that occur when light from a microscope interacts with an inhomogeneous scattering layer, such as a skull when viewing underlying brain tissue. The corrections undo the scattering to focus the image of the underlying tissue.

## Introduction

Optical microscopy has been crucial for many important insights in biomedical research. However, when light travels through biological tissues, its interaction with cells and other structures causes scattering, which worsens with depth^1^. For this reason, conventional optical microscopy is typically limited to depths of a few hundred micrometres. Confocal and two-photon (2P) microscopy^2^ can mitigate some of the scattering and enable an increased depth penetration by using only ballistic photons for focusing. However, the exponential decay of ballistic light with imaging depth still limits these approaches to <100 µm for confocal microscopy, <1 mm for 2P microscopy and <2 mm for three-photon microscopy^1,3,4^.

The interaction of light with inhomogeneous media has been studied under two different regimes. First, in the regime of optical aberrations, large-scale variations of the refractive index lead to a general mild deterioration of the imaging quality^5^. Second, as the spatial range of the refractive index inhomogeneities approaches the wavelength of light, the interaction is dominated by scattering^6^. In this case, illuminating a turbid medium with a coherent light source leads to the generation of a speckle pattern, in which the wavefront appears scrambled. Yet, information is not lost because there is a linear relationship between optical wavefronts at the input plane and the output plane of a scattering medium^7–11^. Thus, both aberrations and scattering can in principle be inverted and compensated for. Techniques based on this idea include modular adaptive optics^12–14^, wavefront sensing^15–17^, pupil segmentation techniques^18,19^ and iterative optimization^20,21^. Most of these techniques are best suited for either the aberration or the scattering regime but not for both. To flexibly correct both scattering and aberrations in vivo, we recently developed a technique termed focus scanning holographic aberration probing (F-SHARP)^22^. F-SHARP measures the amplitude and phase of a point-spread function (PSF) inside a medium, which contains both high- and low-angle scattering information. Because F-SHARP is not limited to the refresh rate of the wavefront-shaping element, it enables the use of a high-pixel-count spatial light modulator (SLM) and allows correction for a large (>1000) number of modes at high speeds.

Despite many advances in wavefront shaping over the last decade, it has still not become a widely used method for in vivo imaging in biomedical research. The reasons for this slow adoption are as follows: first, the limited FOV that one can obtain with conventional wavefront shaping; second, the usually very complicated setups required for the implementation of wavefront corrections on top of a nonlinear microscope.

The lateral range across which a correction pattern will still lead to an increase in image quality (the corrected FOV) depends on the thickness and the scattering properties of the sample. This lateral range is linked to the correlations that light exhibits when propagating through inhomogeneous media^23–27^. These correlations, also known as the “tilt/tilt” and “shift/shift” memory effect, have been combined with wavefront shaping^28–31^ to increase the corrected fields of view, but in the case of thick scattering media, the improvement was moderate and limited to a few µm.

There are imaging scenarios in which a single dominant scattering layer can be considered mostly responsible for the deterioration of the image quality (e.g., imaging underlying tissue through a thin, strongly scattering skull or imaging stars through the atmosphere). In these cases, one can increase the corrected FOV by directly imaging the wavefront shaper onto this scattering layer (as opposed to the objective pupil, which is commonly done). This approach, termed conjugate adaptive optics, was first applied in astronomical imaging. Astronomers achieved an expansion of the corrected FOV by combining lenses and physically displacing the wavefront shaper within the optical train of the system^32–35^. Conjugate adaptive optics have also been applied to optical microscopy, where the wavefront shaper is translated within the optical path of the microscope^36–38^. This implementation of conjugate adaptive optics makes it difficult to quickly scan the focal plane across different imaging depths because it requires physical translation of the wavefront shaper.

Here, we demonstrate a novel conjugate wavefront compensation scheme in which the conjugation of the correction pattern against the dominant scattering layer is achieved by translating the correction pattern against the back aperture of the microscope objective. Such an implementation offers the benefit of versatile depth conjugation without the need for moving the wavefront-shaping element within the optical path. Moreover, we present an implementation of conjugate F-SHARP in a single optical module that can be attached to virtually any home-built or commercial nonlinear microscope. We utilise this implementation of conjugate F-SHARP to deliver high-resolution images with an increased FOV and perform in vivo imaging through a thinned skull preparation in mouse lines with both sparse and dense labelling of cellular fluorescence.

## Principle

### Conjugate F-SHARP with a stationary wavefront shaper

**Fig. 1:**
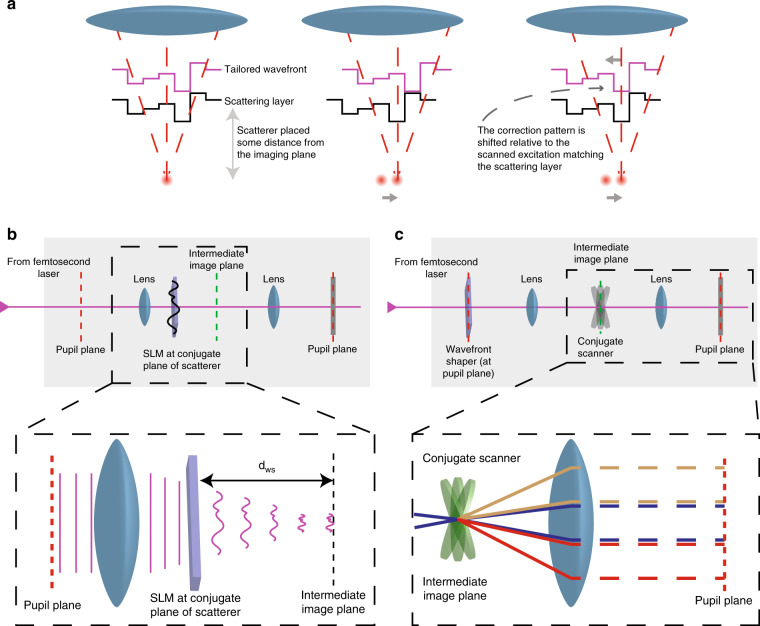
Pupil vs conjugate wavefront correction and z-scanless implementation. **a** In adaptive optics/scattering compensation scanning microscopy, a correction pattern is applied to the excitation beam, which undoes the scattering induced by the inhomogeneous medium between the objective and the imaging plane (left). If the dominant scattering layer is positioned at a considerable distance away from the imaging plane compared with the wavelength, the correction pattern ends up getting scanned against the layer itself (middle). Although part of the information for the compensation is still there, no efficient scattering compensation is achieved. We can compensate for this effect by descanning the correction pattern against the scattering layer (right). **b** One approach to overcoming this problem is to directly image the wavefront shaper at the position of the dominant scattering layer between the imaging and the pupil plane. However, in this case, correcting for different distances of the scattering layer to the imaging plane requires the physical displacement of the wavefront shaper within the optical path. **c** Alternatively, we achieve the proper conjugate wavefront correction by actively scanning the correction pattern against the pupil of the optical system by a 2D scanning mirror introduced in the intermediate imaging plane. Correcting for different distances between the scattering layer and the imaging plane is achieved by changing the relative displacement of the correction pattern against the pupil aperture.

In a raster scanning imaging system, a cone of light emerges from the objective lens and converges towards the imaging plane to form the desired focus spot. If the system is telecentric, as in most modern microscopy systems, scanning the focus spot across the image plane results in a lateral displacement of the light cone relative to the object. If the focus is aberrated by a scattering layer in the sample, one can recruit any of the multiple adaptive optics techniques to calculate the corresponding wavefront pattern that will enhance the image quality. The wavefront is modulated in such a way that when it encounters the scattering layer, it will undo the aberrating/scattering effect and will lead to a sharp focus at the centre of the imaging plane (Fig. 1a, left). The problem that arises in this context is that in a pupil conjugate adaptive optics scheme, the shaped wavefront is scanned against the phase aberration together with the original light cone, leading to an imperfect correction at positions away from the centre of the image (Fig. 1a, middle).

One can overcome this problem by ensuring that the compensating wavefront remains superimposed against the scattering layer, irrespective of the focus position within the image plane^36^ (Fig. 1a, right). Mertz et al.^36^, Park et al.^37^ and Tao et al.^38^ have accomplished this by shaping the wavefront at a plane conjugate to the position of the dominant scattering layer (Fig. 1b). This approach, termed conjugate adaptive optics, requires physical translation of the wavefront shaper within the optical path as the distance between the image plane and the scattering plane changes.

The diffraction of a tight focused spot is quickly dominated by far-field diffraction as it moves away from the focal plane^39^. Therefore, we can approximate the relationship between the wavefront at the imaging location and the wavefront at the plane of the scattering medium by means of a Fourier transform given that the scattering layer is at a certain distance away from the image plane. Considering that the back aperture of the microscope objective (the pupil plane) is also linked to the image plane via a Fourier transform, we conclude the following: we can approximate a translation of the wavefront at the scattering layer plane by a translation at the pupil plane. This insight offers an alternative implementation of conjugate adaptive optics, where the constant superposition of the scattering compensating wavefront is achieved by the scheme shown in Fig. 1c. A pair of *X*–*Y* tilting mirrors can be placed after the wavefront-shaping element at the Fourier plane of the pupil, such that the tilt of the mirrors leads to a translation of the correction pattern. Thus, we can achieve a virtual conjugation of the wavefront shaper to different depths by controlling the displacement of the correction pattern relative to the back aperture of the microscope objective. A more detailed description of this principle, together with an analytic expression of the relationship between the position of the scattering layer and the voltage control of the secondary mirrors, is given in the Supplementary material.

### Modular implementation of F-SHARP

Adaptive optics and wavefront correction hold much promise for the study of deep tissues, but the adoption of these techniques in biomedical research has been limited by the complexity of their implementation. To overcome this hurdle, we propose a modular and compact technical implementation of conjugate F-SHARP. The new module is versatile and adaptable to virtually any pre-existing 2P microscope.

The principle of F-SHARP microscopy is based on the reconstruction of the amplitude and phase of the scattered E-field PSF inside the scattering medium. The appropriate wavefront correction that leads to the compensation of aberrations and scattering is the complex conjugate of the Fourier transform of the reconstructed PSF at the image plane. We can separate the method into three distinct core components: (1) a wavefront-shaping element optically conjugated to the back focal plane of the microscope objective, (2) a secondary scanning mechanism for scanning the aberrated weak beam against the strong beam and (3) a way to change the relative phase between the two beams.

For the implementation of conjugate scanning, we included an extra pair of galvanometric mirrors placed at the intermediate image plane to enable the transverse translation of the SLM relative to the back aperture of the microscope objective (Fig. 1c). A detailed description of the technical implementation of conjugate F-SHARP is presented in the Supplementary material.

## Results

**Fig. 2:**
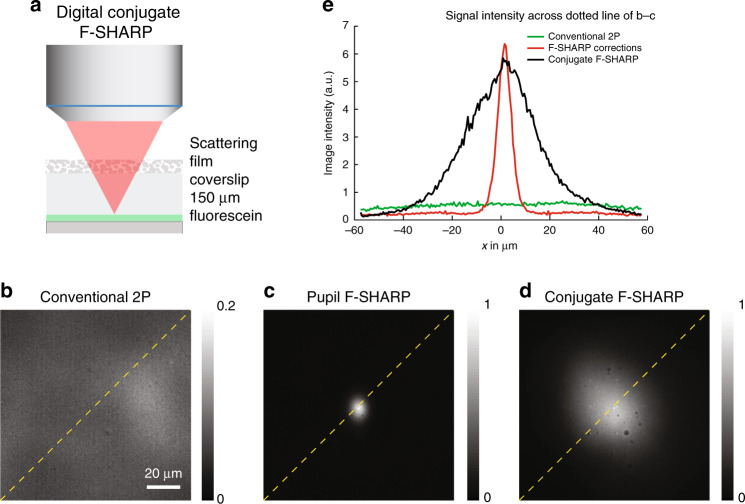
Conjugate F-SHARP of a uniform fluorescein sample through a thin scattering layer. Imaging comparison of a uniform fluorescein layer placed 150 μm away from a thin (≲50 µm) scattering layer: (**a**) conventional 2P microscopy (**b**), pupil F-SHARP imaging (**c**) and conjugate F-SHARP (**d**). Intensity profile of the fluorescence image captured with the three configurations along the dotted line of **b**–**d** (**e**). Images in **b** through **d** were acquired with the same laser power and pixel dwell time and normalized to the maximum value of the 3.

To characterize conjugate F-SHARP and verify its ability to increase the corrected FOV, we imaged a film of fluorescein placed 150 μm under a thin scattering layer (Fig. 2a). We first captured images of the fluorescent layer with a conventional 2P microscope (Fig. 2b) and compared them against the pupil corrected image (Fig. 2c) and the conjugate F-SHARP image (Fig. 2d). Based on the cross-section of the images (Fig. 2e), we observed that pupil F-SHARP increases the image intensity by more than tenfold (at the position of the correction), but delivers a relatively limited FOV (FWHM = 10 μm) centred around the original correction location at the centre of the image. Conjugate F-SHARP, implemented by continuously translating the correction pattern against the back focal plane of the microscope objective, allowed us to extend the corrected FOV to almost 40 μm, an increase of four times for a scattering layer placed only 150 μm away from the imaging plane.

**Fig. 3:**
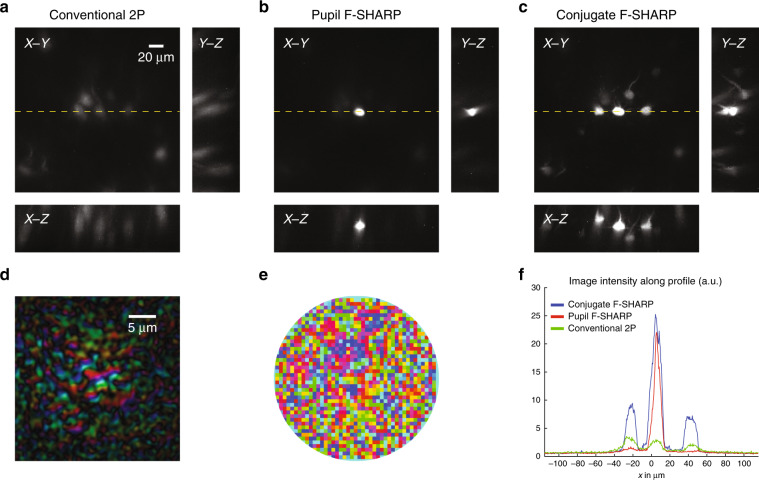
Imaging through the thinned skull of a sparsely labelled THY1-YFP mouse 470 μm below the brain surface. Maximum intensity projection of a 190 × 190 × 80 μm^3^ volume using conventional 2P (**a**), pupil F-SHARP (**b**) and conjugate F-SHARP (**c**). Images in **a**–**c** are presented on the same colour scale and with a saturation corresponding to 0.5 of the maximum signal. The measured E-field PSF at the centre of the image (**d**) and the corresponding correction pattern assigned to the wavefront shaper (**e**) demonstrate a PSF that is highly scattered (phase shown in pseudo colour, using a circular HSV colour map). The conjugate F-SHARP module allows us to increase both the signal intensity and the resolution over an extended FOV compared with conventional 2P and F-SHARP. The achieved FOV increases from 15 μm (pupil F-SHARP) to 80 μm (**f**).

Having established that conjugate F-SHARP can deliver an increased corrected FOV through thin scattering layers, we explored the capability of the method in vivo by imaging cortical excitatory neurons in anaesthetized mice. We imaged layer V cortical neurons sparsely expressing a yellow fluorescent protein (YFP) in a Thy1-YFP transgenic mouse^40^ through a thinned skull preparation. We acquired images of a 190 × 190 × 80 μm^3^ volume and presented them as a maximum intensity projection (Fig. 3a–c). Utilizing F-SHARP, we could reconstruct the E-field PSF through the skull at the centre of the FOV (Fig. 3d) and apply the corresponding wavefront correction to the SLM (Fig. 3e). Conventional 2P microscopy (Fig. 3a) delivered a very low-resolution, noisy image, where the somata of a few neurons could be identified. Pupil-conjugated F-SHARP (Fig. 3b) enhanced the image quality but only at the centre of the FOV, where the correction was calculated (~10-fold increase in the 2P image intensity). Conjugate F-SHARP, however (Fig. 3c), delivered an enhanced image over an extended FOV of 80 μm (Fig. 3f), with similar enhancement to that of pupil F-SHARP.

**Fig. 4:**
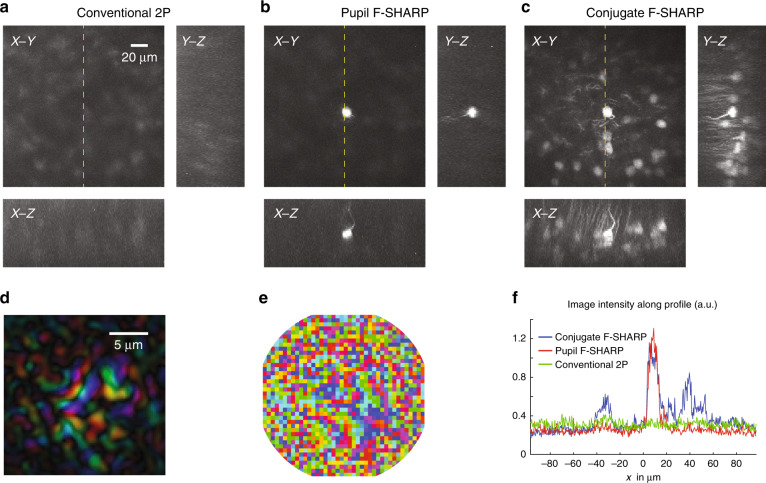
Imaging through the thinned skull of a densely labelled Rbp4-Cre/Ai9 mouse 360 μm below the brain surface. Maximum intensity projection of a 190 × 190 × 80 μm^3^ volume using conventional 2P (**a**), pupil F-SHARP (**b**) and conjugate F-SHARP (**c**). As in Fig. 3, the images in **a**–**c** are presented on the same colour scale, and saturation corresponds to 0.5 of the maximum signal. The measured E-field PSF at the centre of the image (**d**) and the corresponding correction pattern assigned to the wavefront shaper (**e**) demonstrate a scattered PSF with a smaller number of modes compared to Fig. 3. The high variability in the skull thinning process can lead to these differences. The conjugate F-SHARP module allows us to increase both the signal intensity and the resolution over an extended FOV compared with conventional 2P and F-SHARP (**f**).

We also verified the capabilities of conjugate F-SHARP to obtain high-quality 2P images from a mouse brain, in which neurons were densely labelled with a red fluorescent protein, tdTomato. We imaged through the thinned skull of a Rbp4-Cre/Ai9 transgenic mouse in vivo. We captured a volumetric imaging dataset with conventional 2P microscopy, with pupil F-SHARP corrections and conjugate F-SHARP (Fig. 4). We present the maximum intensity projection along the three axes from the three imaging modalities in Fig. 4a–c, respectively. We used the measured E-field PSF at a location at the centre of the FOV (Fig. 4d) to reconstruct the corresponding phase map that would cancel the scattering (Fig. 4e). Comparing the three imaging modalities, we observe that the conventional 2P microscope fails to capture the morphology of the neurons within the sample (Fig. 4a, f). Pupil F-SHARP corrections deliver an increased resolution and image intensity (an ~5-fold enhancement of the 2P signal intensity), which was, however, limited only to the area around the location where the wavefront correction was calculated (Fig. 4b, f). Employing conjugate F-SHARP allowed us to achieve an increased image intensity (~4.5-fold enhancement of the 2P signal intensity) and considerably better resolution across almost the entire FOV along the transverse as well as the longitudinal directions (Fig. 4c, f).

## Discussion

We introduced an approach for rapid and versatile conjugate scattering compensation. We accomplished this by translating the correction pattern against the back aperture of the microscope objective rather than by physically moving the wavefront-shaping element within the optical path. We used the technique to image through both sparsely and densely expressing neurons within an intact mouse brain through a thinned skull preparation.

We achieved an ~8-fold increase in the corrected FOV (~80 μm FOV for conjugate F-SHARP) when imaging a Thy1-YFP mouse at 470 μm deep inside the brain, with a corresponding ~10-fold enhancement of the 2P intensity. The FOV increase through the thinned skull of the Rbp4-Cre/Ai9 mouse was higher (10-fold), albeit with a relatively smaller enhancement of the 2P intensity (5-fold and 4.5-fold for pupil and conjugate F-SHARP, respectively). The use of F-SHARP and conjugate F-SHARP led to increased correction in the axial direction (as seen from the *X*–*Z* and *Y*–*Z* maximum intensity projections in Figs. 3 and 4). Further improvement of the axial range was demonstrated by Paudel et al.^41^, who physically translated the wavefront shaper within the optical path of the optical microscope.

In conjugate adaptive optics, we aim to properly match the position of the correction pattern against the scattering layer while performing raster scanning. The ratio between the displacement of the correction pattern against the back aperture of the microscope objective is required to be equal to the ratio between the displacement of the focus spot over the illuminating cone on the scattering medium (Fig. 1 and Supplementary material). From this, we can also derive that the scanning frequency of the displacement of the correction pattern has to be equal to the raster scanning frequency of the microscope. This implies that the galvo scanners that are chosen for the conjugate F-SHARP module have to be able to keep up with the speed of the galvanometric mirrors of the 2P microscope. We implemented the F-SHARP module in conjunction with a 2P microscope, utilizing a pair of 1 kHz galvanometric mirrors, and we chose similar galvos for the module. In the case of a resonant scanning 2P microscope operating at higher line frequencies, the conjugate F-SHARP module would have to contain a resonant mirror as well.

Conjugate F-SHARP, as with other conjugate scattering compensation techniques, is best suited to scenarios where the dominant part of scattering originates from a thin layer (such as the skull). In the case of a thick scattering medium, the scanning range of the corrected PSF is limited by the volume scattering that the light experiences inside the medium. As has been shown by Osnabrugge et al.^27^, these media exhibit a combination of the “tilt/tilt” and the “shift/shift” memory effect, which can be described by the so-called generalized optical memory effect. The corrected FOV in this case can be increased if the appropriate phase pattern is conjugated at a certain depth inside the thick scattering medium. Using conjugate F-SHARP, we can dynamically achieve the conjugation of the scattering compensating wavefront at any depth. The technique can prove powerful for thick scattering media of varying thickness across the FOV.

The use of phase-only liquid crystal SLMs for wavefront shaping is usually linked with optical setups that exhibit decreased power efficiency. Here, we presented the design and implementation of an optical system based on SLM wavefront corrections and F-SHARP similar to reference^22^, where the optical implementation imposes minimal loss to the beam power due to the double pass through a polarizing beam-splitter (Supplementary material, Section 1). Moreover, minimizing the distance that the separated beams travel independently (between the beam-splitter and the SLM/MEMS mirror) greatly increases the phase stability of the system. At the same time, a phase stepping scheme that relies on an electro-optic modulator can be driven at very fast rates. In the experiments presented here, phase stepping was performed at every pixel of the acquired image. Fast phase stepping leads to better performance of the system because the rejection of laser fluctuation noise can enhance the accuracy of the reconstructed wavefront^19^.

In summary, the combination of a versatile conjugation scheme with a modular and adaptable design increases the reach of wavefront shaping and deep tissue microscopy to greater fields of view.

## Materials and methods

### Surgical procedures

All experiments were performed according to protocols approved by the Berlin Animal Ethics committee (Landesamt für Gesundheit und Soziales, LAGeSo) and complied with the European animal welfare law.

Five-week-old Thy1-YFP^40^ or Rbp4-Cre/Ai9 transgenic mice were anaesthetized with 1.5% isoflurane and tested for lack of tail pinch reflex. Their eyes were protected with ointment (Visidic, Bausch+Lomb), and their body temperature was kept at 37 °C with a feedback sensor system composed of a rectal temperature probe and a heating pad (DC Temperature controller, FHC). Surgical tools were heat-sterilized using a glass bead steriliser (Steri 250, Keller, Fine Science Tools), and all surfaces were wiped with a 70% ethanol solution prior to surgery. An incision of ~1 cm was performed above the midline, and connective tissue was carefully removed to expose the skull. Further cleaning was performed using a microcurette (Fine Science Tools) to remove any remaining tissue on the skull. The coordinates of the primary somatosensory cortex were marked, and the skull was washed thoroughly with Ringer solution (in mM: 135 NaCl, 5 KCl, 5 HEPES, 1.8 CaCl_2_ and 1 MgCl_2_). After drying the skull carefully and avoiding the zone of interest, a needle (25G) was used to mark the surface of the skull to increase the contact surface with the glue and strengthen the bond with the lightweight metal head implant placed on the contralateral hemisphere of the zone of interest. Cyanoacrylate glue (Loctite 401) or UV-curing adhesive (OptiBond, Kerr) was applied to the exposed surface of the skull, avoiding the region of interest. Next, a recording chamber was built from dental cement covering the head implant and going all around but not covering the imaging area. Before the dental cement dried completely, the walls of the recording chamber were carefully shaped and then filled with Ringer’s solution and thoroughly cleaned. Once the skull was dry, we used a drill head (500 µm diameter, Komet, Brassler) operated by a dental drill (Success 40, Osada) to thin the skull over the area of interest (square of 3 mm × 3 mm). The thinning process was carried out in short drilling periods of 30 s followed by washing with Ringer’s solution to remove bone dust and cool the thinned surface. This process was repeated until the blood vessels were clearly visible, which corresponded to a final bone thickness of ~50 µm^42^. Next, a small drop of cyanoacrylate glue was applied to the thinned skull to secure a 3 mm × 3 mm glass coverslip over the area for imaging. Finally, the skull was covered with Ringer’s solution, and the mouse was transferred to the 2P setup.

### Scattering film

For the experiments presented in Fig. 2, we dissolved fluorescein dye in water and deposited a small droplet of the fluorescent solution on a glass slide. We placed a #1 glass coverslip (mean thickness of 150 μm) on top and sealed the sample. On top of the coverslip, we placed a layer of Parafilm with a thickness of 125 μm, using the Parafilm as a scattering layer.

## Supplementary information

Supplementary Information
