# Supplementary material for: Dynamic conjugate F-SHARP microscopy
Source: Light Sci Appl. 2020 Jun 30;9:110. doi: 10.1038/s41377-020-00348-x (PMC7326995; doi:10.1038/s41377-020-00348-x)
Supplement: Supplementary file 1 — Supplementary Information [file 41377_2020_348_MOESM1_ESM.pdf]

Dynamic conjugate F-SHARP microscopy – Supplementary information

Ioannis N. Papadopoulos[1], Jean-Sebastien Jouhanneau[2], Naoya Takahashi[3], David Kaplan[3], Matthew Larkum[3], James Poulet[2], and Benjamin Judkewitz[1]

[1.] Einstein Center for Neurosciences, NeuroCure Cluster of Excellence, Charité – Universitätsmedizin Berlin, Charitéplatz 1, 10117 Berlin, Germany

[2.] Max Delbrück Center for Molecular Medicine, Robert-Rössle-Str. 10, 13092 Berlin, Germany

[3.] Institute for Biology, Humboldt University, Charitéplatz 1, 10117 Berlin, Germany

1. Detailed description of conjugate F-SHARP module

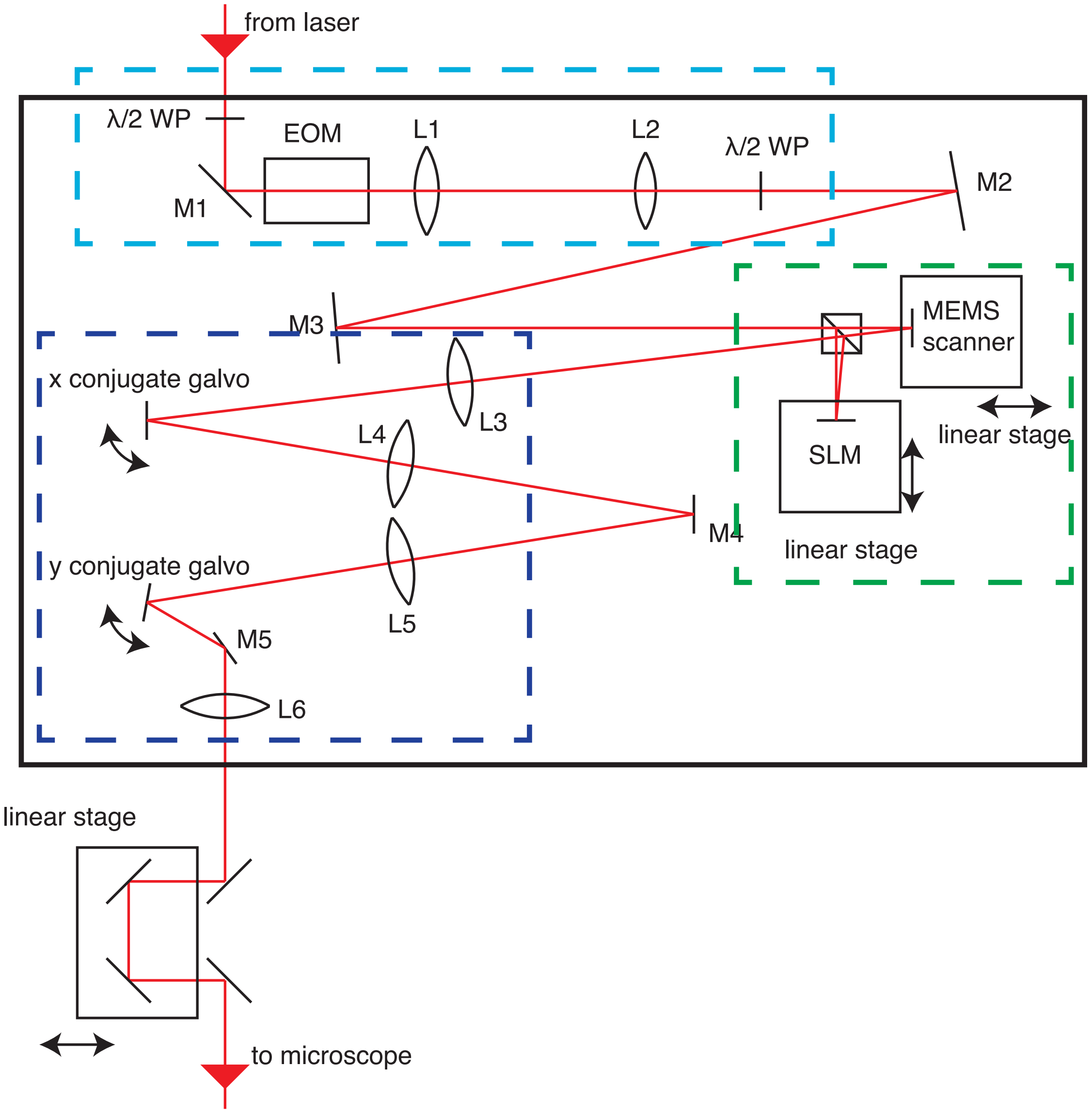

Figure S1. Detailed schematic of optical path of conjugate F-SHARP module. λ/2 WP - half waveplate, L1 through L6 - lens, M1 through M5 - mirror, SLM - spatial light modulator.

In this section we present a detailed technical description of the conjugate F-SHARP module. The goal of such an implementation is to be easily applicable to a wide range of different 2P microscope. The only prerequisite for the installation of the module in a 2P microscope is optical access to its entrance pupil, which in scanning microscopes usually coincides with the location of the galvanometric scanning mirrors.

As has been presented in[1], F-SHARP is based upon the following concept. We split the excitation beam into two beams, one which stays stationary within the field of view (FOV) while the second gets scanned against it. We step the relative phase between the two beams and can therefore infer the complex amplitude

of the PSF inside the scattering medium. Assigning a different intensity to the scanning and stationary beams allows us to reconstruct the amplitude and phase of the scattered electric field PSF (E-field PSF) inside the turbid medium. Based on the time-reversal property of light propagation, we can use the complex amplitude of the inferred E-PSF to calculate the appropriate wavefront correction that will undo the scattering and lead to the formation of a sharp focus spot inside the scattering medium. We repeat the above correction steps a limited number of times (usually 3), until the reconstructed wavefront converges. After the wavefront correction has been identified, the correction pattern is applied onto the SLM, the secondary beam is blocked and we can acquire images similar to any other scanning microscopy technique.

We designed the conjugate F-SHARP system presented here with the intent of placing all the optical components into a module with a single input and output beam. For a simpler description we can conceptually split the optical implementation of the module in 3 separate parts. As shown in Fig. S1, the light blue box on the top, contains all the components responsible for the control of the power ratio and the phase modulation between the two beams. The optical elements within the green box take care of the splitting, relative scanning, wavefront corrections and subsequent recombination of the two beams. Finally, the dark blue box contains the optomechanical components that implement the conjugate functionality. We adjust the beam diameter between the different submodules to the appropriate size needed by the optical components used. Having provided a general picture about the implementation we proceed below in a more detailed description.

The output of a femtosecond laser source, (Spectra Physics MaiTai, central wavelength λ = 920 nm, pulse duration < 100 fs, and corresponding bandwidth of 12nm) passes through a $\lambda/2$ waveplate (AHWP05M-980 Thorlabs) that controls the power ratio between the vertical (stationary) and horizontal (scanning) polarization of the input beam. The beam goes through an amplitude EOM (Thorlabs EOM-AM-NR-C2) which in this case is used to change the relative phase between the two polarizations depending on the voltage applied (detailed information on the phase stepping scheme can be found in Section 2 of the Supplementary Material). We set the beam diameter of the input beam to 1 mm so that it does not suffer clipping from the EOM aperture. We then magnify the beam to a diameter of 5 mm using the 4F telescope (L1, AC254-40-B, L2 AC254-200-B) so that it fills the aperture of the secondary scanner. The beam is reflected off the mirrors M2 and M3 and directed to the polarizing beam-splitter. The horizontal polarization is reflected off the secondary scanner (Mirrorcle, 5mm diameter MEMS mirror) while the vertical polarization bounces off the Spatial Light Modulator (Meadowlark, XY512). Both the MEMS mirror and SLM surface are slightly tilted relative to the input so that the reflected beams can be separated from the incoming one. The SLM and MEMS mirror are placed on translation stages that allow us to control and match the temporal overlap between the two beams. The two polarizations are then recombined using the same polarizing beam splitter. Tilting the SLM and MEMS mirror surface by a small angle enables the beam to be slightly displaced relative to the input. This allows the use of the same polarization beam splitter both for the splitting as well as the recombination, increasing the power throughput of the optical system.

The two beams enter the third and final submodule (dark blue box in Fig. S1) which implements the conjugate adaptive optics capability. As described in the Principle Section of the manuscript, the conjugation of the wavefront correction is achieved by the appropriate displacement of the SLM pattern against the back aperture of the microscope objective. The two galvo mirrors are placed at the intermediate image plane that is formed by the lenses L3 and L5. A tilt of the galvo mirrors will therefore result in a shift of the correction pattern against the pupil of the microscope objective. The final lens L6, transforms the image plane to the pupil plane and projects it onto the final scanning mirrors of the 2P microscope (not shown here).

To aid the alignment of the system and the correct placement of the SLM plane onto the primary scan mirrors of the 2P microscope we implement a double mirror piston that allows us to change the optical path between the last lens and the plane of the primary galvos. The magnification of the telescope comprised by lenses L3-L4 is set to one while the L5-L6 telescope is set to 1.25. The final beam size on the primary galvo mirrors of the microscope is therefore ~ 6 mm.

Finally, the beams go through a linear polarizer that projects the two orthogonal polarizations into a single one causing them to interfere. We can control the final power ratio between the scanning and stationary beam by the combination of the first waveplate and the linear polarizer.

For all the experiments presented in the main manuscript, we used the Nikon CFI 16x, NA = 0.8, water immersion objective. We set the beam diameter equal to the size of the back aperture of the microscope objective therefore utilizing the full NA of the objective for the imaging experiments.

2. Phase stepping using an AM-EOM

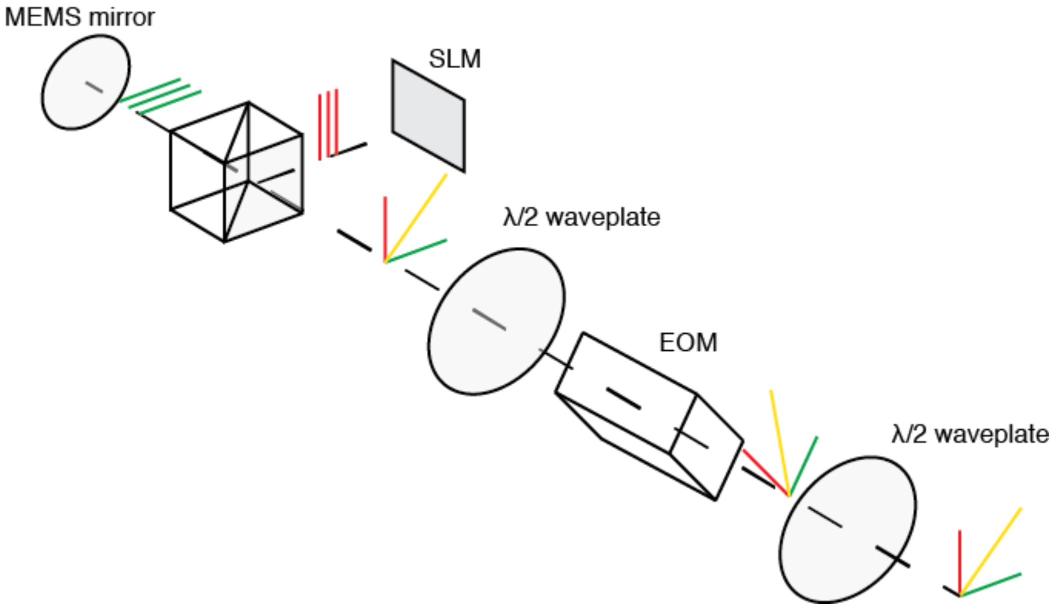

Figure S2. Fast phase stepping using an electro-optic modulator. A linearly polarized input beam can be decomposed into two orthogonal linear polarization states. Matching the orthogonal polarization states with the crystal

axes of the amplitude EOM we can apply a phase difference between the two beams. The first waveplate rotates the input polarization state to the 45° orientation of the AM-EOM crystal. The second waveplate restores the polarization to the original frame of reference.

One of the crucial steps in F-SHARP is the phase-stepping between the scanning (weak) and the stationary (strong) beams. We achieve that by separating the input light into two polarizations, each one associated with one of the scanning (horizontal) and the stationary (vertical) beams. We use an amplitude electro-optic modulator as a phase stepping device between the two beams of F-SHARP. In particular we used a Thorlabs EOM (Thorlabs EOM-AM-NR-C2) where the ordinary and extraordinary axes are rotated by 45° relative to the horizontal plane. To achieve a pure phase modulation, we first direct the beams through a half waveplate that rotates the input polarization so that each one of the scanning and stationary beams are aligned with one of the axes of the EOM. Changing the voltage that is applied on the EOM we can assign a phase difference between the two beams. The two polarizations then propagate through another half waveplate that restores their rotation to the original frame of reference. The two polarizations finally pass through a polarizing beam-splitter cube that splits them. The horizontal polarization is reflected off of the MEMS mirror - therefore being the scanning beam during the estimation of E-PSF, while the vertical one bounces off the wavefront shaping device. After their respective reflection, the two beams recombine through the same polarizing beam-splitter cube.

In order to separate the incoming beam from the outgoing one, we assign a small angle on both the MEMS mirror and the SLM so that the two beams end up spatially separated at a distance away from the reflection. Finally, similar to the previous implementation of F-SHARP, to enforce the interference of the two beams, they propagate through a polarizer. The two orthogonal polarization thus project on the same axis of the polarizer and interfere.

3. Reconstruction of correction patterns

In F-SHARP we reconstruct the amplitude and phase of the scattered E-PSF inside the turbid medium by spatially scanning a weak copy of the scattered PSF against a strong stationary version of the PSF in two dimensions. This two-dimensional raster scanning is performed in the same manner as in a regular laser scanning microscope. The spatial extent of the scanning (the FOV in imaging terms) is defined by the opening angle of the secondary scanning mirror. Each acquired image contains a number of lines (typically 100) with a number of samples within each line (typically 100). The acquired pixel size (given by the lateral field of view over the pixel number) defines the spatial resolution of the reconstructed PSF. The maximum size of the pixel needs to be equal to half the wavelength of the light so that no information is lost during acquisition. On the other hand, the lateral extent of the measured PSF will singularly define the sampling at the pupil plane. One needs to capture the full extent of the scattered PSF which in turn will provide a reconstruction with the maximum number of modes that can be corrected.

In the performed experiments we acquired a 100 x 100 pixels image of the complex PSF inside the medium. The scanning angle of the secondary scanning mirror was set such that the captured FOV was 30.3 by 30.3 μm^2 for the experiments presented in Fig. 3 and 20.8 by 20.8 μm^2 for those in Fig. 4. We calculate the number of modes contained within each correction pattern by taking the ratio between the area of the back aperture and the mean mode area. The mean mode area is calculated from the full width to half maximum of the autocorrelation of the correction pattern. The correction pattern in Fig. 3 contains ~ 360 modes, while the correction pattern in Fig. 4 ~ 132 modes. The scanning rate of the secondary mirror defines the time needed for the acquisition of a single image of the scattered PSF. In our experiments we used a MEMS mirror with a scanning rate of 500 Hz, which allows us to capture a single image within 200 ms. In total, we acquired 4 phase-shifted images of the E-PSF for a single measurement of the PSF inside the medium, amounting to a total of 800 ms of acquisition time for each measurement.

Scattering compensation at a single location within the FOV does not rely on the scattering properties of the medium (biological tissue, mouse cortex in the demonstrated experiments). As we elaborated on our previous paper[1], F-SHARP measures the transmission matrix of the medium to the chosen position within the FOV and based on the principle of optical phase conjugate can efficiently undo the scattering. This will allow us to form a sharp focus spot at this position of the FOV behind the scattering medium, in reflection.

On the other hand, the ”corrected FOV” or “isoplanatic patch” will depend on the properties of the scattering medium and the so-called memory effect. This memory effect can either be of a tilt-tilt or shift-shift nature. The tilt-tilt (or angular) memory effect can be observed in thin scattering media with its range being inversely proportional to the thickness of the medium. The shift-shift (or translation) memory effect can be observed in directionally scattering media. At the same time, Osnabrugge et al.[2] have proposed a more generalized theory of the memory effect where a medium can exhibit a combination of both.

Based on the above, we can reach the conclusion that the correction pattern that will effectively undo the scattering for the measured position within the FOV is directly given by the Fourier transform of the measured E-PSF. This Fourier transform equivalence is a direct outcome of the principle of optical phase conjugate and the optical properties of lenses and does not depend of the properties of the scattering medium (thick or thin, weak or strong scattering). At the same time, imaging deeper inside the brain (through thicker scattering tissue) will cause this correction pattern to decorrelate faster as we attempt to image areas around the original correction position therefore reducing the memory effect and limiting the corrected FOV.

4. Illumination of the SLM and effects on image correction

Both in pupil and conjugate aberration compensation techniques, the correction pattern is calculated for a single position within the FOV. When the scattering can be attributed to a single dominant scattering layer at a distance away from the imaging plane, the calculated correction pattern corresponds to a probing of

this scatterer within the cone of the original excitation beam. We demonstrate that in Supplementary Figure S3, where the mapped region of the scatterer is the one contained within the initial excitation cone. No information about the scatterer can be inferred around this original position (gray region of scatterer in Fig. S3 left). However, when we start imaging locations around the original position where the correction was calculated for, the excitation beam will start encountering areas of the scatterer that have not been mapped by the original calculation pattern. If the excitation beam overfills the back-aperture of the microscope objective, this portion of the illumination will encounter the scatterer without any correction and will lead to a scattered contribution to the PSF on top of the corrected one. This scattered contribution to the PSF will decrease the quality of the image by adding background noise.

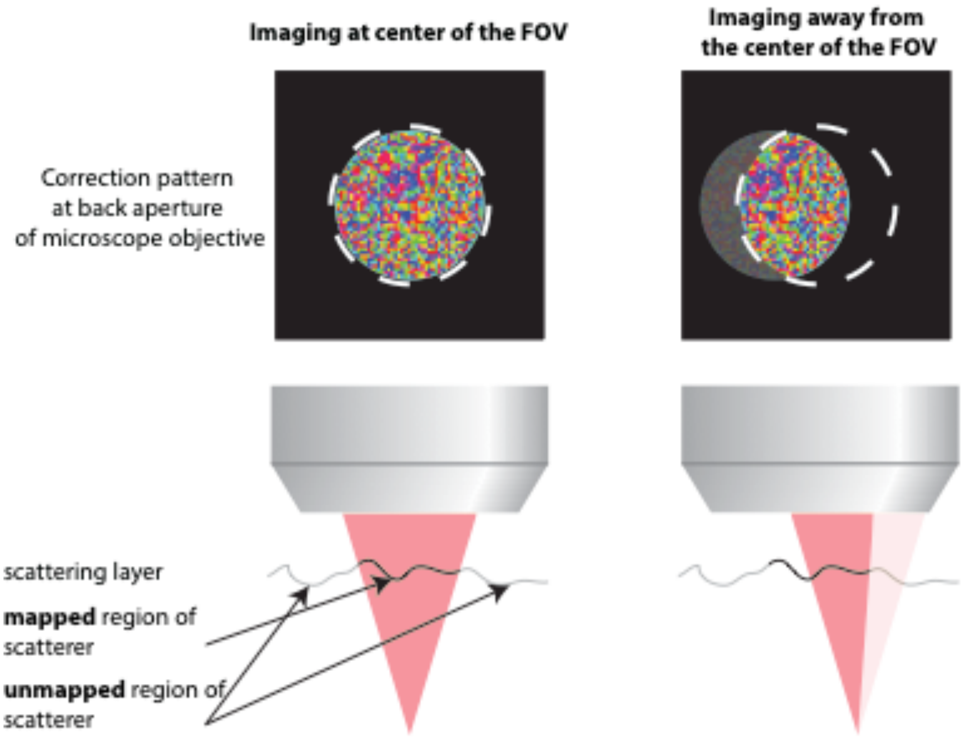

Figure S3. Effect of the correction pattern translation to the illumination. The correction pattern assigned on the SLM is calculated for a specific position within the FOV and completely fills the microscope objective back aperture. In conjugate F-SHARP, when imaging a position away from the center, the correction pattern is shifted relative to the back aperture in order to properly conjugate the corrections with the scattering layer. In this configuration, with the illuminating beam being equal in size with the pupil size, the part of the back aperture that corresponds to the "unmapped" areas of the scattering layer are not illuminating. Such an implementation reduces the noise caused by an uncorrected beam going through the areas of the scattering layer that are not compensated for.

In conjugate F-SHARP we can set the size of the excitation beam to be equal to the correction pattern in the SLM (equal to the aperture of the back-aperture of the microscope objective). In this case, at a different

scanning position within the FOV, the correction pattern will be correctly superimposed with the “mapped” region of the scatterer while at the same time, the “unmapped” region will not be illuminated at all.

5. Focus spot translation at image plane vs translation of the correction pattern against the back aperture

In a telecentric imaging system, translating a focus spot in the image plane implies that the cone of light that creates this focus spot is translated by an equal amount against the front element of the microscope objective. This in turn means that the cone of light will be shifted against the scattering sample by an equal translation of the focus spot within the FOV. For proper scattering compensation at the new position, the modulated wavefront that encounters the scattering layer needs to be shifted by an equal amount in the opposite direction (Figure S4). The ratio between the shifted distance against the scatterer (Δx_S) and the size of the diameter of the illuminating cone on the scatterer (w_S), is equal to the ratio between the translation of the correction at the back aperture over the diameter of the back aperture. Therefore, we have that $\frac{\Delta x_{\mathrm{S}}}{\mathrm{w_S}} = \frac{\Delta x_{\mathrm{BA}}}{\mathrm{w_{BA}}}$. From Figure S4, we can see that the diameter of the illumination beam on the scatterer is $\mathrm{w_S} = 2 * \mathrm{d_S} * \tan\theta_{\mathrm{inc}}$, and $\theta_{\mathrm{inc}} = \sin^{-1}\mathrm{NA}$.

In a scanning microscope, the position of the excitation beam within the FOV is approximately proportional to the deflection angle of the galvo mirrors and therefore to the control voltage of the galvo mirrors, $\Delta x_S = \alpha * V_{\mathrm{galvo}}$ with α being a scaling factor. Similarly, the translation of the correction pattern against the back-aperture of the microscope objective is proportional to the deflection angle (control voltage) of the x and y conjugate galvo mirrors, $\Delta x_{\mathrm{BA}} = \beta * V_{\mathrm{conj.\,galvo}}$. Putting all the above together we have,

$$V_{\mathrm{conj.\ galvo}} = \frac{a}{\beta} * \frac{\mathrm{w_{BA}}}{2 * \mathrm{d_S} * \tan\left(\sin^{-1}\frac{\mathrm{NA}}{\mathrm{n_{IM}}}\right)} V_{\mathrm{galvo}}$$

Ultimately the above relationship gives us the relative scaling that needs to be applied to the conjugate galvo voltage (dependent on the NA and the distance of the scattering layer to the image plane) as a function of the voltage control of the spot scanning voltage. This translation will enforce the correct overlap of the correction pattern with the scattering layer.

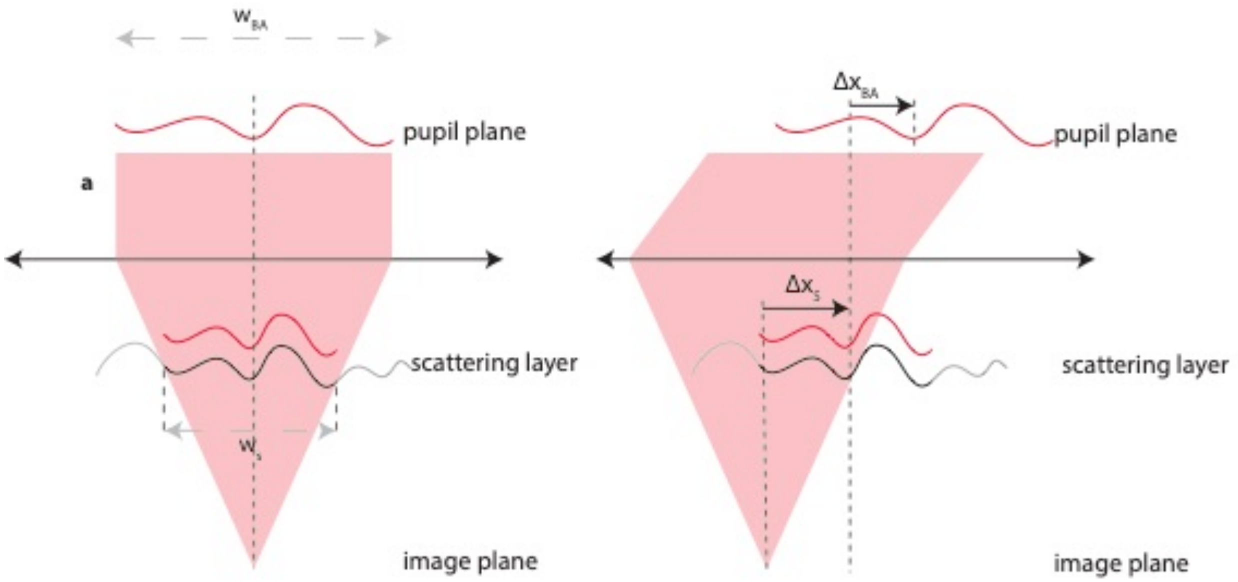

Figure S4. Relative translation of the correction pattern as a function of the displacement of the focus. The relative translation of the correction phase map against the back-aperture that will enforce the overlap of the correction pattern against the scattering layer at every position of the scanning is proportional to the displacement of the focus. The signal that controls the conjugate galvo deflection is therefore a scaled version of the scanning galvo signal.

References

1. Papadopoulos, I. N., Jouhanneau, J.-S. bastien, Poulet, J. F. A. & Judkewitz, B. Scattering compensation by focus scanning holographic aberration probing (F-SHARP). *Nat. Photonics* **11**, 116–123 (2016).

2. Osnabrugge, G., Horstmeyer, R., Papadopoulos, I. N., Judkewitz, B. & Vellekoop, I. M. Generalized optical memory effect. *Optica* **4**, 886–892 (2017).
